# Association between hyperleptinemia and oxidative stress in obese diabetic subjects

**DOI:** 10.1186/s40200-015-0159-9

**Published:** 2015-04-14

**Authors:** Gautam Pandey, Mohamed Sham Shihabudeen, Hansi Priscilla David, Ethirajan Thirumurugan, Kavitha Thirumurugan

**Affiliations:** Centre for Biomedical Research, School of Bio Sciences & Technology, 206, Structural Biology Lab, VIT University, Vellore, India; Palar Hospital, Senthil Clinic & Lab, Gudiyattam, India

**Keywords:** Leptin, Malondialdehyde, Glutathione peroxidase, Superoxide dismutase, Protein carbonyl groups, Type 2 diabetes, Obesity

## Abstract

**Background:**

Obesity is a worldwide metabolic disorder affecting all types of people. The mechanism by which increased body fat mass that leads to insulin resistance and type 2 diabetes is not yet clearly known. There is a possible crosstalk between leptin, an adipokine and insulin signaling. Leptin mediates insulin sensitivity in hepatocytes; however, its concentration has found to be increased in obese and diabetic subjects. These subjects also have high incidence of oxidative stress status. Therefore, knowing the level of leptin present in obese diabetic subjects will be informative along with its relation to oxidative stress.

**Methods:**

A small population study was performed to explore the association between leptin concentration and oxidative stress status in control and obese type 2 diabetic subjects. Oxidative stress status parameters like malondialdehyde (MDA), superoxide dismutase activity (SOD), glutathione peroxidase activity (GSH-Px), and protein carbonyl (PCO) groups content was measured spectrophotometrically in serum of 43 subjects. Serum Leptin concentration was measured by quantikine sandwich ELISA assay.

**Results:**

The strong positive correlation between MDA (malondialdehyde) and leptin in obese diabetic patients (*ρ* = 0.787, *P* < 0.05) suggests close association between lipid peroxidation and hyperleptinemia. In addition, observed positive correlation between protein carbonyl groups and leptin level in obese diabetic subjects (*ρ* = 0.599, *P =* 0.001) suggest that hyperleptinemia might also be associated with increased protein oxidation. In multiple logistic regression analysis, leptin has shown a significant association with obese type 2 diabetes [odds ratio (OR): 1.161, 95% confidence interval (Cl): 1.027-1.312, P < 0.05], but the significance is lost after adjusting for Age, BMI, MDA and anti-oxidant parameters.

**Conclusions:**

In the subjects with both obesity and diabetes, there is a significant degree of association between hyperleptinemia and oxidative stress. This association reinforces the existing understanding that obese subjects who also have diabetes are vulnerable to cardiovascular complications driven by increased oxidative stress and hyperleptinemia.

## Background

Obesity and diabetes, together called as diabesity, is the ever growing metabolic disorder of industrialized and developing countries. At present, India stands next to China with 63 million diabetic population [1]. The mechanism linking insulin resistance, oxidative stress, adipokines in type 2 diabetes and obese subjects is poorly understood. There is strong evidence that adipocytes play a critical role in metabolism through the secretion of hormones and cytokines that alter whole-body energy homeostasis [2]. Leptin, 16-kDa protein secreted by adipocytes was first identified as the product of a gene designated *ob* (obese) in laboratory mice [3,4]. In adipocytes, leptin inhibits glucose uptake, impairs lipogenesis, glycogen synthase and inhibits lipolysis [5]. In hepatocytes, leptin triggers insulin-like effects through regulating insulin-signaling pathway [6]. Hence, there is a possible cross talk between leptin and insulin signaling, making hepatocytes more sensitive to insulin. Interestingly, presence of higher leptin levels especially in obese subjects makes them even more resistance to insulin-like effects thus mediating type 2 diabetes in obese subjects. This has been well confirmed in studies showing rise in circulating leptin levels in relation to BMI and percentage total body fat [7,8]. Leptin levels were found to be either same or lower in subjects with diabetes but significantly higher in obese and obese diabetic condition [9,10]. Being an adipokine, leptin levels rise with increasing BMI and body fat, but its relation with diabetes and insulin resistance seems to be intriguing, as individuals who have both obesity and type 2 diabetes exhibit higher leptin levels when compared to obese or diabetic population alone. The mechanism linking this behavior may be contributed in part to oxidative stress. Hyperleptinemia and oxidative stress are the consequences of obesity [11], and may play a role in mediating T2DM in obesity. Studies have shown leptin as a possible mediator of oxidative stress in obese and obese diabetic subjects. Leptin has been shown to correlate strongly with MDA (product of lipid peroxidation) levels in T2DM subjects [10]. Leptin also increases the reactive oxygen species (ROS) in endothelial cells [12]. In fact endothelial dysfunction is one mechanism which can disturb glucose homeostasis via increased oxidative stress [13]. Oxidative stress plays a critical role in the development of type 2 diabetes and associated cardiovascular complications [14,15]. Interestingly administration of leptin was shown to mitigate the expression levels of genes related to oxidative stress and inflammation in the skeletal muscle of ob/ob Mice [16]. Leptin treatment was also shown to reduce lipid peroxidation in STZ-diabetic rats through enhancing the GSH levels [17]. This shows the necessity of leptin in reducing the oxidative stress in muscle tissues and the possible inverse oxidative stress inducing effect due to aberrations in leptin signaling in hyperleptinemia. Also in South Indian population, there is lack of data to clearly explain the relationship between leptin and oxidative stress. Therefore it is of imperative need to analyze the association of hyperleptinemia and oxidative stress in the population with diabetes and obesity. In the present study, we have investigated the possible relationship of leptin with various oxidative stress parameters in obese diabetic and healthy individuals. The serum leptin levels are compared with age, Body Mass Index (BMI), Waist to Hip ratio (WHR), systolic/diastolic blood pressure (BP), Fasting Blood Glucose (FBG), Malondialdehyde (MDA), Superoxide dismutase (SOD), Glutathione peroxidase (GPx) and Protein carbonyl (PCO) content in these groups.

## Methods

### Subjects

A total of 43 subjects from Gudiyattam, Tamilnadu, India were included in the study. Two groups were selected: control population (n = 17) containing non-obese, non-diabetic subjects and obese type 2 diabetic population (n = 26) containing subjects suffering from both obesity and type 2 diabetes. The criteria for diabetes were based upon WHO/IDF reports on definition and diagnosis of diabetes mellitus [18]. The criteria for obesity were re-defined according to the WHO guidelines which use a BMI of 25 kg/m^2^ for defining obesity in South Asians [19]. Intra-abdominal obesity was also assessed by measuring waist-to-hip ratio (WHR) which existed if WHR ≥ 1.0 for males and ≥ 0.86 for females [20].

The individuals were asked to reveal their age, gender, blood pressure and other risk factors including arterial hypertension, smoking, hyperlipidemia, current medication and other socioeconomic variables by replying to a series of standard questionnaire. Patients who had experienced clinical infection of renal, hepatic or any malignant disease, myocardial infarction, surgery or major trauma were excluded from the study.

The study was carried out according to the ethical standards of Palar hospital, Gudiyattam and VIT University, Vellore. Written informed consent was obtained from all the individuals participated in the study.

### Sample collection and analysis

Venous blood (5 ml) was collected from each individual after over-night fasting (>10 hr). Serum was then obtained, aliquoted and stored at −20°C for further analysis. Glucometer (One touch Horizon™) was used to analyze fasting blood glucose levels. Serum leptin levels were estimated using Leptin ELISA Kit following the manufacturer’s protocol (DRG Instruments, Germany). Lipid peroxidation was measured by estimating serum malondialdehyde (MDA) levels-a thiobarbituric acid reactive substance formed as a byproduct of lipid peroxidation [21].

The assay of Superoxide dismutase (SOD) activity was done by using the procedure of Kakkar et al. [22]. The assay was measured in terms of inhibition of Nitroblue tetrazolium (NBT) reduction by Superoxide dismutase enzyme present in the serum.

Glutathione Peroxidase (GPx) activity was determined as reported by Rotruck et al. [23]. The procedure is based upon a reaction in which the leftover glutathione (GS-SG) present in the serum (after glutathione peroxidase converts monomeric glutathione to glutathione disulphide) reacts with the DTNB (5,5′- dithiobis 2-nitrobenzoic acid) to form a compound which absorbs at 412 nm. The Protein Carbonyl (PCO) group was used as a marker of severe protein oxidation. It involves the derivatization of the carbonyl group with 2,4-dinitrophenylhydrazine (DNPH), which leads to the formation of the stable dinitrophenyl hydrazone product [24].

### Statistical analysis

The results were expressed as mean ± S.D. Two-tailed Student’s *t*-test is employed to determine the significance of the data. Pearson correlation was used to examine the strength of correlation between various risk factors and Leptin levels. Multiple logistic regression analysis was performed using leptin as independent variable and type 2 diabetes and obesity as dependent variable after adjusting for Age, BMI, and oxidative stress parameters. The values were considered as statistically significant if *p* < 0.05.

## Results

Anthropometric measures, clinical and oxidative stress parameters, and leptin concentration of the two populations are presented in Table 1. The level of leptin and MDA is significantly high in obese diabetic population compared to control subjects. Antioxidant defense status as indicated by SOD and GPx activity also decreases in obese diabetic population with SOD showing significant reduction. Protein oxidation as represented by PCO has shown a significant rise in obese diabetic population as compared to control population.

**Table 1:** **Clinical, oxidative stress status, and leptin levels of the study groups**

**Parameter**	**Control (non-obese, non-diabetic) population**	**Obese type 2 diabetic population**	**P-value**
Total subjects	n = 17	n = 26	
Age (years)	41.2 ± 10.1	55.55 ± 8.15	0.191
BMI (kg/m2)	23.40 ± 2.6	28.57 ± 3.06	0.0002
WHR	0.975 ± 0.09	1.09 ± .07	0.004
Systolic blood pressure (mm/hg)	144.4 ± 23.5	153.7 ± 28.7	0.955
Diastolic blood pressure (mm/hg)	98.4 ± 17.7	88.4 ± 12.69	0.373
FBG (mg/dl)	95.4 ± 12.0	171.23 ± 41.46	0.008
Leptin (ng/ml)	12.03 ± 7.4	26.85 ± 13.11	0.003
MDA (μM)	1.50 ± 0.51	1.99 ± 0.7	0.003
SOD activity (U/ml)	2.99 ± 0.98	1.92 ± 1.19	0.001
GSH Px Activity (U/ml)	0.173 ± 0.01	0.16 ± 0.02	0.818
Protein carbonyls’ content (μM/Litre)	8.99 ± 0.06	10.71 ± 3.9	0.0007

Data are expressed as mean ± SD, p < 0.05 is considered as statistically significant for all the groups compared to control.

To analyze the influence of oxidative stress parameters on leptin levels, Pearson correlation is performed (Table 2). The strong correlation between BMI and leptin levels in both the population (control: r = 0.646, *P* < 0.01; obese diabetic: r = 0.806; P < 0.001) has clearly shown the effects of BMI on leptin concentration. There is a significant strong correlation exist between MDA and leptin levels in obese diabetic population (r = 0.787, *P* = <0.001). Antioxidant defense status (SOD and GPx activity) has shown negative correlation and protein oxidation (PCO content) has shown positive correlation with leptin levels in obese diabetic subjects.

**Table 2:** **Correlation between leptin concentrations and the parameters of oxidative stress status**

**Variables**	**Control (non-obese, non-diabetic) population (n = 17)**		**Obese diabetic population (n = 26)**	
	**Pearson’s coefficient**	***P***	**Pearson’s coefficient**	***P***
Age (years)	−0.624	0.053	0.479	0.243
BMI	0.646	0.005	0.806	0.0001
WHR	−0.819	0.058	0.450	0.302
FBG (mg/dl)	−0.001	0.999	−0.272	0.179
MDA (μM)	0.256	0.322	0.787	0.0001
SOD activity (U/ml)	−0.116	0.657	−0.190	0.353
GPx activity (U/ml)	0.010	0.970	−0.690	0.069
nM/mg	0.249	0.335	0.599	0.001

Pearson Correlation coefficient are indicated, *p* < 0.05 is considered as statistically significant.

In multiple logistic regression model (Table 3), leptin has shown a significant association with obese type 2 diabetes [odds ratio (OR): 1.161, 95% confidence interval (Cl): 1.027-1.312, P < 0.05], but the significance is lost after adjusting for Age, BMI, MDA and anti-oxidant parameters.

**Table 3:** **Multiple logistic regression analysis with obese type 2 diabetes as dependent variable and leptin as independent variable**

**Variable**	**Odds ratio (OR)**	**95% confidence interval (Cl)**	***P*** **-value**
Leptin-Unadjusted	1.161	1.027-1.312	0.017
Adjusted for Age	1.110	0.948-1.298	0.195
Adjusted for BMI	0.793	0.341-1.844	0.590
Adjusted for MDA	0.326	0.035-3.050	0.326
Adjusted for SOD	0.449	0.160-1.263	0.129
Adjusted for PCO	1.413	0.763-2.617	0.271

Odds ratio values and p values are indicated, p < 0.05 considered as statistically significant for all the groups compared to control.

## Discussion

This study aims to explore the possible association of leptin with oxidative stress status in normal and obese type 2 diabetic population. It has clearly presented the following case: Leptin is positively correlated with BMI in both the population. Leptin is positively correlated with lipid peroxidation and protein oxidation in obese type 2 diabetic population. Leptin is also a strong predictor of obese type 2 diabetes with BMI, WHR and oxidative stress status.

Leptin is an adipokine known to regulate energy expenditure and keep body fit and healthy [25]. On the contrary, biologically it is also an active mediator of type 2 diabetes and so its exact role in the development of insulin resistance still remains unclear [26]. In our study, obese diabetic individuals have significantly higher levels of circulatory leptin. The same group is also characterized by reduced antioxidative activity, increased lipid peroxidation, and higher protein oxidation as indicated by SOD/GSH-Px, MDA and PCO levels. This strongly indicates the role of metabolic stress on circulatory leptin levels.

MDA, a major toxic TBARS (Thiobarbituric acid reactive substances) product were measured in our volunteers as an index of lipid peroxidation. It has been shown that hyperleptinemia plays a key role in the formation of lipid peroxides thus mediating oxidative stress, corroborated by the previous observation that leptin increased the oxidative stress in tissues where there is high rate of fatty acid oxidation, including muscle tissues [27]. In results of correlation analysis (Table 2) leptin is strongly correlated with MDA in both the population suggesting an association of lipid peroxidation and leptin. This association is even greater for obese diabetic individuals indicating that these people are under severe oxidative stress.

There is a general understanding that BMI is a strong predictor of obesity. But WHR (Waist to Hip ratio) is also a useful indicator of intra-abdominal obesity. Our data, however, confirmed that BMI alone may be a strong predictor of clinical manifestations of obesity as a strong and significant correlation exists between leptin and BMI in obese diabetic population.

To evaluate the antioxidant potential of human subjects against the superoxide radical, we have measured *in vitro* SOD activity and correlated with serum leptin levels. The SOD activity is significantly decreased in obese diabetic condition compared to healthy subjects (Table 1). This agrees with the earlier report of low SOD activity in type 2 diabetic patients [28]. Glutathione peroxidase has also been assessed for its peroxidase activity in serum, i.e., to reduce free hydrogen peroxide to water. Our results have also shown a decrease in the peroxidase activity of the enzyme in obese diabetic subjects (Table 1). However, we failed to establish a significant relationship between leptin, SOD and GSH-Px in the correlation analysis.

Another important marker of oxidative stress is protein carbonylation measured through estimating protein carbonyl groups content (PCO) in serum. Measuring PCO group is advantageous over other biomarkers of oxidative stress due to their early formation and detectable stability arising from protein side chains (Pro, Arg, Lys, and Thr). It has been shown that PCO level increases in diabetic patients as compared to healthy volunteers [29]. We also observed a significant increase in protein oxidation in the patient group as compared to normal healthy group (Table 1). In fact, PCO levels show a strong and significant correlation with leptin among obese diabetic individuals, making them vulnerable to the effects of hyperleptinemia.

Thus we have observed a close association between hyperleptinemia and oxidative stress in obese diabetic subjects. Previous finding on leptin induced oxidative stress via increased fatty acid oxidation corroborates this observation [30]. Obese diabetic subjects are more vulnerable to cardiovascular complications further driven by increased oxidative stress and hyperleptinemia [31]. It is hard to say without any *in vivo* model that whether it is hyperleptinemia which induces oxidative stress in obese subjects and makes them susceptible to insulin resistance or is it oxidative stress which is causing an abrupt increase in leptin, thus mediating its harmful effects. Since leptin seems to be more of a protective hormone [32], we can speculate that may be oxidative stress induced hyperleptinemia causes an aberrant leptin signaling (leptin resistance), thus rendering leptin from its protective effects and inducing insulin resistance in obese people.

Hence, our current study on a small Indian population warrants the need for the prospective studies to understand the association between oxidative stress and hyperleptinemia.

## Conclusions

In the subjects with both obesity and diabetes, there is a significant degree of association between hyperleptinemia and oxidative stress. This association reinforces the existing understanding that obese subjects who also have diabetes are vulnerable to cardiovascular complications driven by increased oxidative stress and hyperleptinemia.

## References

[1] Whiting DR, Guariguata L, Weil C, Shaw J (2011). IDF diabetes atlas: global estimates of the prevalence of diabetes for 2011 and 2030. Diabetes Res Clin Pract.

[2] Fruhbeck G, Gomez-Ambrosi J, Muruzabal FJ, Burrell MA (2001). The adipocyte: a model for integration of endocrine and metabolic signaling in energy metabolism regulation. Am J Physiol Endocrinol Metab.

[3] Zhang Y, Proenca R, Maffei M, Barone M, Leopold L, Friedman JM (1994). Positional cloning of the mouse obese gene and its human homologue. Nature.

[4] MacDougald OA, Hwang CS, Fan H, Lane MD (1995). Regulated expression of the obese gene product (leptin) in white adipose tissue and 3 T3-L1 adipocytes. Proc Natl Acad Sci U S A.

[5] Müller G, Ertl J, Gerl M, Preibisch G (1997). Leptin impairs metabolic actions of insulin in isolated rat adipocytes. J Biol Chem.

[6] Zhao AZ, Shinohara MM, Huang D, Shimizu M, Eldar-Finkelman H, Krebs EG (2000). Leptin induces insulin-like signaling that antagonizes cAMP elevation by glucagon in hepatocytes. J Biol Chem.

[7] Maffei M, Halaas J, Ravussin E, Pratley R, Lee G, Zhang Y (1995). Leptin levels in human and rodent: measurement of plasma leptin and ob RNA in obese and weight-reduced subjects. Nat Med.

[8] Considine RV, Sinha MK, Heiman ML, Kriauciunas A, Stephens TW, Nyce MR (1996). Serum immunoreactive-leptin concentrations in normal-weight and obese humans. New Engl J Med.

[9] Okumura T, Taniguchi A, Nagasaka S, Sakai M, Fukushima M, Kuroe A (2003). Relationship of regional adiposity to serum leptin level in nonobese Japanese type 2 diabetic male patients. Diabetes Metab.

[10] Ajala MO, Ogunro PS, Idogun SE, Osundeko O (2009). Relationship between Plasma Antioxidant Status and Leptin in Controlled and Non‐Controlled Type 2 Diabetic Non‐Obese Women. Int J Endocrinol Metabol.

[11] Stefanović A, Kotur-Stevuljević J, Spasić S, Bogavac-Stanojević N, Bujisić N (2008). The influence of obesity on the oxidative stress status and the concentration of leptin in type 2 diabetes mellitus patients. Diabetes Res Clin Pract.

[12] Yamagishi SI, Edelstein D, Du XL, Kaneda Y, Guzmán M, Brownlee M (2001). Leptin induces mitochondrial superoxide production and monocyte chemoattractant protein-1 expression in aortic endothelial cells by increasing fatty acid oxidation via protein kinase A. J Biol Chem.

[13] Hu FB, Stampfer MJ (2003). Is type 2 diabetes mellitus a vascular condition?. Arterioscler Thromb Vasc Biol.

[14] Lipinski B (2001). Pathophysiology of oxidative stress in diabetes mellitus. J Diabetes Complications.

[15] Kuyvenhoven J, Meinders A (1999). Oxidative stress and diabetes mellitus: Pathogenesis of long-term complications. Eur J Intern Med.

[16] Sáinz N, Rodríguez A, Catalán V, Becerril S, Ramírez B, Gomez-Ambrosi J et al. Leptin administration downregulates the increased expression levels of genes related to oxidative stress and inflammation in the skeletal muscle of ob/ob mice. Mediat inflamm 2010. doi:10.1155/2010/784343 10.1155/2010/784343 PMC2910527 20671928

[17] Gülen Ş, Dinçer S (2007). Effects of leptin on oxidative stress in healthy and Streptozotocin-induced diabetic rats. Mol Cell Biochem.

[18] World Health Organization. Definition and diagnosis of diabetes mellitus and intermediate hyperglycemia. Report of a WHO/IDF Consultation, 2006: 1-46.

[19] Anoop Misra A, Shrivastava U (2013). Obesity and Dyslipidemia in South Asians. Nutrients.

[20] BMI OC (1998). Clinical guidelines on the identification, evaluation, and treatment of overweight and obesity in adults.

[21] Yagi K (1987). Lipid peroxides and human diseases. Chem Phys Lipids.

[22] Kakkar P, Das B, Viswanathan PN (1984). A modified spectrophotometric assay of superoxide dismutase. Indian J Biochem Biophys.

[23] Rotruck J, Pope A, Ganther H, Swanson A, Hafeman D, Hoekstra W (1973). Selenium: biochemical role as a component of glutathione peroxidase. Science.

[24] Dalle-Donne I, Rossi R, Giustarini D, Milzani A, Colombo R (2003). Protein carbonyl groups as biomarkers of oxidative stress. Clin Chim Acta.

[25] Hwa JJ, Fawzi AB, Graziano MP, Ghibaudi L, Williams P, Van Heek M (1997). Leptin increases energy expenditure and selectively promotes fat metabolism in ob/ob mice. Am J Physiol.

[26] Segal K, Landt M, Klein S (1996). Relationship between insulin sensitivity and plasma leptin concentration in lean and obese men. Diabetes.

[27] Solinas G (2010). Leptin Signalling Coordinates Lipid Oxidation with Thermogenesis and Defence Against Oxidative Stress. Clin Exp Pharmacol Physiol.

[28] Kesavulu M, Rao BK, Giri R, Vijaya J, Subramanyam G, Apparao C (2001). Lipid peroxidation and antioxidant enzyme status in Type 2 diabetics with coronary heart disease. Diabetes Res Clin Pract.

[29] Pandey KB, Mishra N, Rizvi SI (2010). Protein oxidation biomarkers in plasma of type 2 diabetic patients. Clin Biochem.

[30] Yamagishi SI, Edelstein D, Du X-l, Kaneda Y, Guzmán M, Brownlee M. Leptin induces mitochondrial superoxide production and monocyte chemoattractant protein-1 expression in aortic endothelial cells by increasing fatty acid oxidation via protein kinase A. J Biol Chem. 2001;276(27):25096–100. 10.1074/jbc.M007383200 11342529

[31] DeFronzo RA, Ferrannini E (1991). Insulin resistance: a multifaceted syndrome responsible for NIDDM, obesity, hypertension, dyslipidemia, and atherosclerotic cardiovascular disease. Diabetes Care.

[32] Balasubramaniyan V, Shukla R, Murugaiyan G, Bhonde RR, Nalini N (2007). Mouse recombinant leptin protects human hepatoma HepG2 against apoptosis, TNF-alpha response andoxidative stress induced by the hepatotoxin-ethanol. Biochim Biophys Acta.

